# Indoor Volatile Organic Compound Exposure Patterns and White Blood Cell Count in Korean Adults: Effect Modification by Smoking

**DOI:** 10.3390/toxics14030225

**Published:** 2026-03-06

**Authors:** Yang Jee Kim

**Affiliations:** College of General Education, Chung-Ang University, 84 Heukseok-ro, Dongjak-gu, Seoul 06974, Republic of Korea; yangjee4@cau.ac.kr

**Keywords:** volatile organic compounds (VOCs), urinary VOC metabolites, source-oriented exposure, systemic inflammation, white blood cell count, smoking, KNHANES

## Abstract

Volatile organic compounds (VOCs) are ubiquitous environmental pollutants, and mixed VOC exposure has been linked to systemic inflammation. However, evidence remains limited regarding source-oriented VOC exposure patterns and their associations with inflammatory biomarkers in the general population. Using data from 1812 Korean adults participating in the Korea National Health and Nutrition Examination Survey (KNHANES) from July 2020 to August 2021, we identified source-oriented urinary VOC exposure patterns through factor analysis, yielding combustion-dominant and solvent-dominant indices. Environmental relevance was evaluated using an airborne VOC index, and associations with white blood cell (WBC) count were examined using generalized linear models, including interaction analyses by smoking status (defined specifically as conventional cigarette users). Both urinary indices were significantly associated with the airborne VOC index (*p* < 0.05), supporting their environmental validity. In models without interaction terms, the solvent-dominant index was positively associated with WBC count (β = 0.091, *p* = 0.030), while the combustion-dominant index did not reach statistical significance (β = 0.107, *p* = 0.081). However, significant interactions by smoking were observed for both indices (*p* for interaction < 0.001). Among conventional smokers, higher exposure to both combustion-dominant β = 0.614, *p* < 0.001) and solvent-dominant β = 0.571, *p* < 0.001) patterns was significantly associated with increased WBC counts, whereas no such associations were found among non-smokers. These findings indicate that while VOC patterns impact systemic inflammation, the associations are significantly modified by cigarette smoking. Our results underscore the importance of source-oriented approaches and the explicit evaluation of effect modification when assessing the health impacts of mixed VOC exposure.

## 1. Introduction

Volatile organic compounds (VOCs) are ubiquitous environmental pollutants originating from diverse sources, including combustion processes, industrial emissions, building materials, household products, and tobacco smoke [1,2]. In indoor environments, VOC exposure is of particular concern because individuals spend the majority of their time indoors, resulting in continuous low-level exposure rather than episodic high-dose events [1,3]. Accumulating evidence suggests that such chronic exposure may contribute to adverse health outcomes through oxidative stress and systemic inflammatory pathways [4].

Urinary metabolites of VOCs are widely used biomarkers of internal exposure, as biological monitoring integrates exposure across multiple routes and reflects internal dose rather than ambient concentrations alone [5]. This approach has been widely applied in both environmental and occupational settings to characterize VOC exposure [6]. Large population-based studies, particularly those using NHANES data, have demonstrated that urinary VOC metabolites capture mixed exposure patterns and exhibit secular trends consistent with changes in ambient and indoor VOC concentrations [7].

Previous biomonitoring studies have also reported substantial differences in urinary VOC metabolite distributions according to smoking status and demographic characteristics, highlighting smoking as a key determinant of internal VOC burden in population-based analyses [8]. These biomarkers have been applied to investigate associations with respiratory diseases, cardiovascular outcomes, metabolic disorders, kidney disease, and dyslipidemia [9,10].

Generally, VOC exposure in real-world settings occurs as complex mixtures rather than as isolated single compounds [2,11]. To address this complexity, recent epidemiological studies have increasingly adopted data-driven approaches such as factor analysis, principal component analysis, or cumulative exposure indices to characterize mixed exposure patterns [7,8]. These approaches suggest that urinary VOC metabolites often cluster in patterns broadly consistent with shared emission sources, with one pattern commonly enriched in metabolites of combustion-dominant compounds (e.g., benzene, acrolein, and 1,3-butadiene) and another enriched in metabolites of aromatic solvents such as xylene and ethylbenzene. However, such groupings reflect predominant associations rather than mutually exclusive source categories. Such source-oriented grouping provides a useful conceptual framework for summarizing mixed VOC exposure patterns [11].

Systemic inflammation has been proposed as a key biological mechanism linking VOC exposure to adverse health outcomes. Experimental and human studies suggest that VOC exposure can induce oxidative stress and modulate inflammatory responses, including changes in inflammatory markers and immune cell activation in respiratory tissues [12,13]. Consistent with clinical evidence, population-based analyses have demonstrated significant associations between VOC exposure and hematological alterations. Specifically, data from NHANES 2005–2010 revealed that elevated blood concentrations of several VOCs are linked to increased white blood cell (WBC) counts, supporting the systemic inflammatory potential of VOC exposure [14]. As an accessible marker of low-grade systemic inflammation, WBC count has also been significantly associated with ambient air pollution in a recent large-scale study of the South Korean population, reinforcing its utility in environmental health assessments [15]. Furthermore, given that elevated WBC counts are established predictors of cardiometabolic risk and long-term mortality across diverse population-based cohorts, they serve as a critical surrogate for evaluating the broader health impacts of environmental pollutants [16,17].

Cigarette smoking is not only a major contributor to combustion-dominant VOC exposure (along with other indoor sources such as incense burning, and gas stove use) but is also strongly associated with elevated WBC counts, reflecting smoking-induced systemic inflammation. Epidemiological studies have consistently demonstrated that combustion-related leukocytosis is at least partially reversible after cessation, supporting a causal inflammatory mechanism [18,19]. Recent evidence from Korean populations has further confirmed the association between smoking and elevated WBC count, as well as other inflammatory markers such as C-reactive protein [20]. Beyond its direct effects, smoking may modify the inflammatory response to environmental pollutants through shared oxidative stress pathways and alterations in immune function [12]. Given that smokers are simultaneously exposed to both tobacco-derived VOCs and environmental VOCs, understanding whether smoking modifies the association between environmental VOC exposure and systemic inflammation is of particular public health importance.

Despite growing evidence, several gaps remain. First, many population-based studies have focused on individual VOC metabolites or total VOC indices without explicitly distinguishing exposure patterns by emission source. Second, relatively few studies have examined source-specific VOC mixtures in relation to systemic inflammation while explicitly evaluating combustion-related effect modification. Third, nationally representative evidence from Korean populations remains limited compared with NHANES-based studies.

To improve conceptual clarity, we distinguished between environmental exposure assessed using airborne VOC concentrations and internal exposure assessed using urinary VOC metabolites. Urinary metabolites were further grouped into solvent-dominant and combustion-dominant patterns using factor analysis. Smoking status was treated as a covariate and effect modifier rather than as a component of the combustion-dominant index.

The objectives of this study were threefold. First, we aimed to identify the source-oriented exposure patterns of urinary VOC metabolites in Korean adults using factor analysis. Second, we sought to validate the environmental relevance of these patterns by examining their associations with indoor airborne VOC measurements. Third, we assessed the associations between VOC metabolite patterns and WBC count as a marker of systemic inflammation, with particular attention to potential effect modification by cigarette smoking status.

## 2. Methods

### 2.1. Study Design and Data Source

This cross-sectional study used data from the Korea National Health and Nutrition Examination Survey (KNHANES), a nationally representative survey conducted by the Korea Disease Control and Prevention Agency (KDCA). The present analysis included data collected between July 2020 and August 2021 as part of the household indoor air quality and environmental hazardous substances biomonitoring survey. We analyzed adults who participated in the environmental hazardous substances biomonitoring component, which includes indoor air quality assessment and urinary VOC biomarker measurements. All data collection and laboratory analyses followed standardized protocols described in the official KNHANES raw data utilization guidelines [21]. This study was conducted as a secondary analysis of publicly available KNHANES data.

### 2.2. Study Population

**Figure 1:**
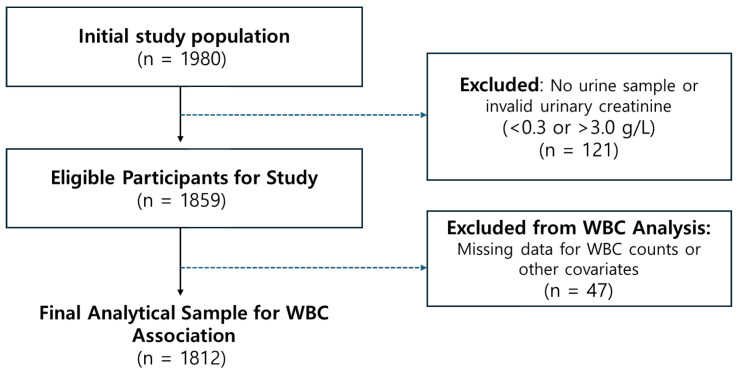
Flowchart of participant selection for the analyses. Participants were excluded sequentially based on the absence of urine samples, invalid urinary creatinine concentrations (<0.3 or >3.0 g/L), and missing outcome or covariate data. The eligible population (N = 1859) was used for demographic characterization (Table 1) and environmental validation analysis (comparing airborne vs. urinary VOCs). After further excluding 47 participants with missing clinical data, the final analytic sample (N = 1812) was used for WBC regression models and interaction analyses.

Among 1980 adults with available urinary VOC metabolite data, 121 participants with invalid urinary creatinine concentrations (<0.3 or >3.0 g/L) were excluded, leaving 1859 eligible individuals. This group was used for demographic characterization (Table 1) and environmental validation analysis. For WBC regression analyses, 47 participants with missing WBC or covariate data were further excluded, resulting in a final analytic sample of 1812 adults. Complete case analysis was used, as the proportion of missing data was small (<3%) and appeared to be missing at random with respect to key variables. The participant selection process is shown in Figure 1.

In this study, smokers were defined specifically as conventional cigarette users based on the KNHANES standard variable (sm_presnt), ensuring a consistent combustion-related VOC exposure profile. Users of only new tobacco products (e-cigarettes) were excluded to maintain the specificity of the analysis.

### 2.3. Urinary VOC Biomarkers

Spot urine samples were collected using standardized KNHANES protocols. Nine urinary VOC metabolites were quantified as biomarkers of internal exposure using validated LC–MS/MS methods implemented by KDCA [21]. Urinary creatinine was measured concurrently to account for urine dilution. For concentrations below the limit of detection (LOD), values were imputed as LOD/√2, a commonly used approach in biomonitoring studies [22].

### 2.4. Construction of VOC Exposure Indices

To characterize mixed VOC exposure patterns, correlation analysis and factor analysis with varimax rotation were applied to log-transformed urinary metabolite concentrations. Factors with eigenvalues greater than 1 were retained, and metabolites with factor loadings ≥0.50 were considered to load strongly on a given factor. Two exposure indices representing predominant source-oriented patterns were constructed by averaging standardized (z-scored) concentrations of metabolites with high loadings on each factor. BPMA was excluded from both indices due to its low communality (0.448) and its distinct industrial exposure profile, which did not align clearly with the predominant patterns identified in the factor analysis.

Airborne solvent-dominant VOC exposure was assessed using the summed concentrations of six indoor air VOCs (benzene, toluene, ethylbenzene, xylene, styrene, and total volatile organic compounds (TVOC)) measured in the KNHANES indoor air quality survey. Formaldehyde, although measured as part of the indoor air quality assessment, was excluded because no corresponding urinary biomarker was available, and PM_2.5_ was not considered because it represents a distinct, non-VOC exposure domain. These six VOCs were selected based on their established metabolic correspondence with the urinary biomarkers included in the factor analysis.

### 2.5. Outcome and Covariates

The primary outcome was white blood cell (WBC) count obtained from the KNHANES health examination component. Covariates selected a priori included age, sex, body mass index, smoking status, alcohol consumption, diabetes status, and indoor carbon dioxide (CO_2_) concentration. Education level and household income were evaluated but excluded from final models because they did not materially alter exposure–outcome associations.

### 2.6. Statistical Analysis

Descriptive statistics were used to summarize participant characteristics and biomarker distributions. Continuous variables were expressed as means and standard deviations, and categorical variables as frequencies and percentages. Differences between groups were assessed using Student’s *t*-test for continuous variables and chi-square tests for categorical variables.

For clarity, indoor airborne VOCs refer to measured concentrations of volatile organic compounds in indoor air. Urinary VOC biomarkers denote log-transformed metabolite concentrations reflecting internal exposure.

Factor analysis was performed on log-transformed urinary VOC biomarkers to identify underlying exposure patterns. Factors with eigenvalues greater than 1 were retained, and varimax rotation was applied to improve interpretability. Factor analysis-derived exposure patterns were interpreted as source-oriented patterns, and corresponding standardized indices were constructed for use in regression models.

The combustion-dominant pattern was interpreted as reflecting exposure to VOCs generated from incomplete combustion processes, including cigarette smoking and other indoor combustion-related activities (e.g., gas stove use and incense burning), based on the predominant metabolite loadings identified in the factor analysis.

Associations between VOC exposure indices and WBC count were evaluated using generalized linear regression models. Regression coefficients (β) and 95% confidence intervals (CI) were estimated. Effect modification by smoking status was examined by including an interaction term (urinary VOC exposure index × smoking status × smoking status) in the regression model. The statistical significance of the interaction was assessed using the Wald test, with *p* < 0.05 considered statistically significant. All models were adjusted for age, sex, body mass index (BMI), smoking status, alcohol consumption, diabetes, and indoor carbon dioxide (CO_2_) concentration.

Statistical significance was assessed using two-sided *p*-values, with *p*-values reported to three decimal places and values < 0.001 reported as *p* < 0.001. Correlation heatmaps and interaction plots were generated using R software. All statistical analyses were conducted using SAS version 9.4 (SAS Institute Inc., Cary, NC, USA) with the PROC GENMOD procedure and R version 4.5.2 (R Foundation for Statistical Computing, Vienna, Austria), following KNHANES analytical guidelines.

Model assumptions were verified through diagnostic procedures, including assessment of residual normality (Q-Q plots and Shapiro–Wilk test), homoscedasticity (residual plots), and multicollinearity (variance inflation factors, VIF). Models were considered valid if VIF values were below 2.5 and residual diagnostics showed no major violations of model assumptions.

## 3. Results

### 3.1. General Characteristics of Study Population

The general characteristics of the study population are presented in Table 1. Among the 1859 participants, 844 (45.4%) were male and 1015 (54.6%) were female, with a mean age of 54.17 ± 17.00 years. Body mass index (BMI) was significantly higher in males than in females (24.73 ± 3.47 vs. 23.42 ± 3.73 kg/m^2^, *p* < 0.0001).

Smoking and alcohol consumption patterns differed markedly by sex. Current smoking was reported by 29.01% of males and 3.36% of females (*p* < 0.0001). Alcohol consumption was more prevalent among males than females (65.76% vs. 37.65%, *p* < 0.0001). The proportion of participants engaging in aerobic physical activity did not differ significantly between males and females (*p* = 0.145). Educational attainment also differed significantly by sex (*p* < 0.0001). Specifically, the proportion of participants with an elementary school education or less was higher in females (24.12%) than in males (12.17%), while a higher percentage of males (43.04%) had a college education or higher compared to females (38.20%).

### 3.2. Correlation Structure Between Indoor Air VOCs and Urinary Biomarkers

**Figure 2:**
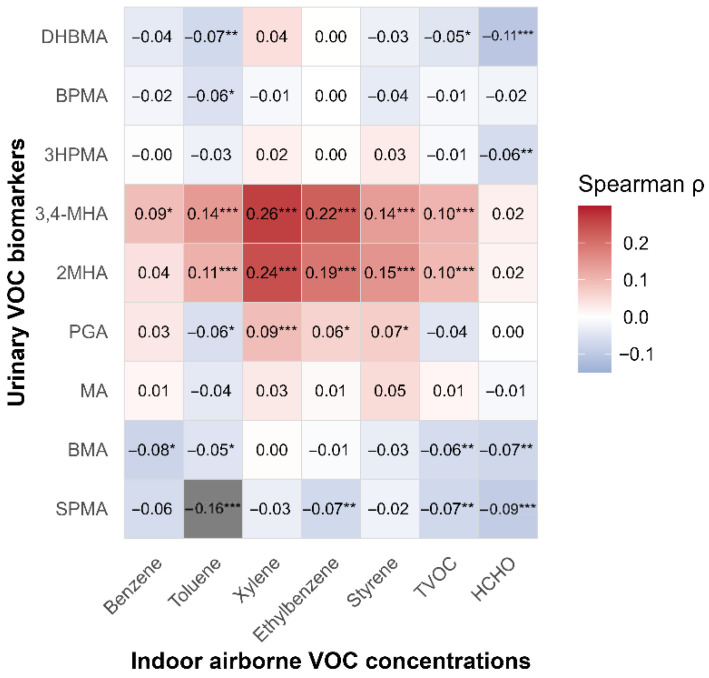
Spearman correlation heatmap illustrating the association structure between indoor airborne VOCs and urinary VOC biomarkers (both log-transformed). Colors indicate the direction and magnitude of correlations (blue, negative; red, positive), with white representing zero correlation. Numeric values within cells denote Spearman’s rho, and asterisks indicate statistical significance (* *p* < 0.05; ** *p* < 0.01; *** *p* < 0.001). The ordering of air substances and biomarkers reflects hypothesized metabolic relationships and mixed exposure patterns rather than strict one-to-one associations.

Figure 2 presents a Spearman correlation heatmap between indoor air VOC concentrations and urinary VOC biomarkers. Correlations were generally weak to moderate but showed distinct clustering patterns. Xylene metabolites (2MHA and 3,4MHA) showed stronger correlations with indoor xylene, supporting their specificity as biomarkers of solvent-dominant exposure, whereas other metabolites (BPMA, DHBMA, 3HPMA) demonstrated broader correlation patterns across multiple VOCs, suggesting mixed or shared exposure sources. These structured correlation patterns are consistent with the source-oriented factor structure identified in Table 2.

### 3.3. Factor Analysis of Urinary VOC Biomarkers

Factor analysis identified two major factors with eigenvalues greater than 1, explaining approximately 28.0% and 27.9% of the total variance, respectively. Together, these two factors accounted for 55.9% of the overall variance in urinary VOC metabolite concentrations (Table 2).

The first factor (Combustion-dominant pattern) was characterized by high loadings for SPMA (0.716), BMA (0.695), DHBMA (0.694), and 3HPMA (0.589). These metabolites are commonly associated with cigarette smoking and combustion-dominant exposures. While MA showed a loading of 0.540 on this factor, its relatively low communality (0.345) suggested that its variance was less well-explained by the shared combustion source compared to the core metabolites.

The second factor (Solvent-dominant pattern) showed dominant loadings for 3,4-MHA (0.943) and 2-MHA (0.928), which are well-established urinary biomarkers of xylene exposure. These metabolites exhibited exceptionally high communalities (0.890 and 0.869, respectively), indicating that this factor strongly represents a distinct solvent-dominant exposure domain. Although PGA exhibited moderate loadings, it was not considered a core contributor due to its low communality (0.298) and lack of clear alignment with a single factor. BPMA was excluded from the final factor structure due to its comparatively lower communality and lack of coherent loading within either dominant factor (see Section 2).

Based on the final factor structure, the combustion-dominant urinary VOC index was defined by SPMA, BMA, DHBMA, and 3HPMA, whereas the solvent-dominant urinary VOC index was defined by 2-MHA and 3,4-MHA. These source-oriented patterns reflect predominant loading structures rather than mutually exclusive exposure categories.

### 3.4. Associations Between Airborne VOC Exposure and Urinary VOC Indices

Table 3 presents the associations between airborne VOC exposure and urinary VOC indices. Airborne VOC exposure was positively associated with both the combustion-dominant urinary VOC index (β = 0.039, 95% CI: 0.004–0.075, *p* = 0.032) and the solvent-dominant urinary VOC index (β = 0.241, 95% CI: 0.189–0.292, *p* < 0.001).

Smoking status showed strong positive associations with both indices, with a substantially higher magnitude observed for the solvent-dominant urinary VOC index (β = 1.295, 95% CI: 1.180–1.410, *p* < 0.001). Age was positively associated with both indices, whereas body mass index showed inverse associations. Indoor carbon dioxide (CO_2) concentration was inversely associated with both indices (*p* < 0.01), while alcohol consumption was not significantly associated with either index after adjustment.

Additional adjustment for education level and household income did not materially alter the association between the airborne VOC exposure index and the solvent-dominant urinary VOC index; therefore, these variables were not retained in the final models.

### 3.5. Association Between Urinary VOC Exposure Index and White Blood Cell Count

As shown in Table 4 the solvent-dominant urinary VOC index was positively associated with WBC count; each one–standard deviation increase was associated with a higher WBC level (β = 0.091, 95% CI: 0.009–0.172, *p* = 0.030). In contrast, the combustion-dominant urinary VOC index showed a positive but non-significant association with WBC count (β = 0.107, 95% CI: −0.013–0.226, *p* = 0.081).

Smoking status was independently associated with elevated WBC levels in both models (Model 1: β = 0.929, 95% CI: 0.694–1.164, *p* < 0.001; Model 2: β = 1.001, 95% CI: 0.784–1.217, *p* < 0.001). Alcohol consumption was inversely associated with WBC count, while diabetes and body mass index (BMI) were positively associated with WBC levels in both models.

### 3.6. Predicted White Blood Cell Levels by Smoking Status Interaction Between Urinary VOC Exposure Indices and Smoking Status in Relation to WBC Count

Table 5 presents the interaction between urinary VOC exposure indices and smoking status in relation to WBC count. Among non-smokers, neither the solvent-dominant index (β = −0.027, 95% CI: −0.117- 0.063, *p* = 0.557) nor the combustion-dominant index (β = −0.023, 95% CI: −0.152–0.106, *p* = 0.728) was significantly associated with WBC count.

In contrast, among smokers, both the solvent-dominant index (β = 0.571, 95% CI: 0.391–0.752) and the combustion-dominant index (β = 0.614, 95% CI: 0.380–0.849) were positively associated with WBC count. The interaction terms between each urinary VOC exposure index and smoking status were statistically significant (*p* for interaction < 0.001 for both indices), indicating that the associations between VOC exposure patterns and WBC count differed significantly according to smoking status.

### 3.7. Predicted White Blood Cell Levels by Smoking Status

**Figure 3:**
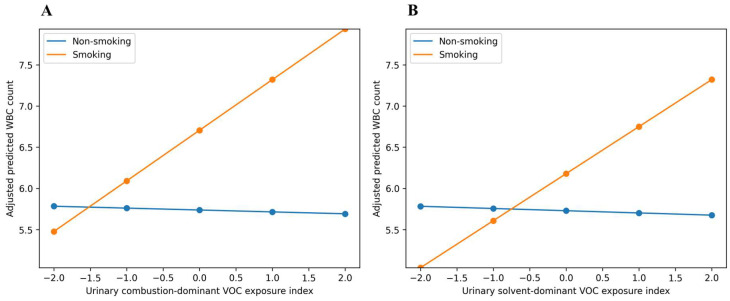
Adjusted predicted WBC count by smoking status across (**A**) urinary combustion-dominant VOC exposure index and (**B**) urinary solvent-dominant VOC exposure index. Predictions were derived from interaction models adjusted for age, sex, BMI, alcohol consumption, and diabetes.

Figure 3 presents adjusted predicted WBC counts according to smoking status across the urinary VOC exposure indices. In both panels, predicted WBC levels were consistently higher among smokers than non-smokers, indicating a clear baseline difference by smoking status.

For the combustion-dominant index (Figure 3A), WBC levels showed little change across index values among non-smokers, whereas a positive gradient was observed among smokers. Similarly, for the solvent-dominant index (Figure 3B), WBC levels remained relatively stable across index values in non-smokers but increased progressively among smokers.

These patterns visually demonstrate that the association between urinary VOC exposure indices and WBC count was predicted to be stronger among smokers than non-smokers, consistent with the significant effect modification by smoking reported in Table 5 (*p* for interaction < 0.001 for both indices).

## 4. Discussion

In this population-based study, we identified two source-oriented urinary VOC metabolite patterns. The solvent-dominant urinary VOC exposure index showed a significant positive association with WBC, whereas the combustion-dominant index showed a positive but non-significant association. Both urinary VOC indices were significantly associated with the airborne VOC exposure index, supporting their relevance as markers of indoor VOC exposure.

The distinction between combustion-dominant and solvent-dominant urinary VOC exposure patterns observed in this study is consistent with the known emission characteristics of combustion- and solvent-dominant compounds reported in previous studies. Combustion-related VOCs are commonly associated with tobacco smoke and fuel combustion, solvent-dominant VOCs, particularly xylene, are commonly emitted from building materials and other indoor consumer products [7,8] and may also be linked to specific occupational environments. Specifically, in the Korean context, indoor xylene concentrations have been identified as major pollutants originating from building materials and adhesives used in residential environments [23]. The environmental relevance of urinary VOC metabolite patterns observed in this study is further supported by previous KNHANES-based analyses demonstrating concordance between indoor airborne VOC concentrations and corresponding urinary biomarkers in the Korean population [24].

Given that individuals spend the majority of their time in indoor environments, solvent-dominant VOC exposure may represent a sustained and pervasive exposure source in the general population, even at relatively low ambient concentrations [3]. Consistent with this, our analysis of airborne pollutants revealed that the airborne VOC exposure index was primarily associated with the solvent-dominant urinary VOC exposure index (β = 0.241, *p* < 0.001) rather than the combustion-dominant urinary VOC exposure index (β = 0.039, *p* = 0.032). This specificity supports our focus on solvent-dominant exposure as a more direct indoor environmental risk factor for systemic inflammation in this study population.”

Accordingly, this source-oriented approach enabled the evaluation of mixture effects while preserving interpretability and avoiding assumptions of direct causal relationships among individual metabolites [11].

Consistent with this interpretation, the solvent-dominant factor identified in our study was predominantly driven by MHA metabolites, supporting the use of an MHA-based index to characterize solvent-dominant VOC exposure. In indoor-dominated exposure settings, urinary biomarkers with high source specificity may provide complementary information to ambient concentration measurements by integrating variability in exposure intensity, duration, and individual metabolic differences [25].

Our findings are consistent with previous biomonitoring studies demonstrating that urinary VOC metabolites cluster according to common emission sources. Konkle et al. [7] reported distinct VOC components corresponding to combustion-dominant (largely driven by smoking) and solvent-dominant sources in NHANES data, and subsequent studies have applied similar multivariate approaches to characterize mixed VOC exposure patterns. Several population-based studies have further linked urinary VOC metabolites to cardiometabolic outcomes, dyslipidemia, kidney disease, and other chronic conditions [9,10].

Although indicators of socioeconomic status, including education level and household income, were evaluated in sensitivity analyses, they did not materially alter the association between the airborne VOC exposure index and the solvent-dominant urinary VOC exposure index. Therefore, these variables were not retained in the final models to improve parsimony. From an exposure assessment perspective, these findings suggest that biomonitoring-based indices may capture aspects of mixed VOC exposure that are not fully reflected by individual ambient concentration measurements, particularly in complex indoor environments [26,27].

To facilitate interpretation of these findings, we distinguished urinary VOC exposure patterns into two analytically meaningful dimensions. One reflects co-exposure with combustion-dominant sources, largely influenced by combustion-related behaviors, whereas the other represents a solvent-dominant exposure pattern more directly linked to airborne VOCs.

The observed association between source-oriented urinary VOC exposure patterns and WBC count is consistent with experimental and toxicological evidence, though the cross-sectional design precludes definitive causal or mechanistic interpretation. Experimental studies suggest that solvent-dominant VOCs may induce oxidative stress and stimulate inflammatory signaling pathways. For example, laboratory investigations have demonstrated that 2-methylhippuric acid (2MHA) and 3,4-methylhippuric acid (3,4MHA)—urinary metabolites of xylene—are associated with markers of oxidative stress and can be linked to pro-inflammatory responses in controlled settings [28]. Although these data support biological plausibility, our cross-sectional findings cannot establish whether the observed associations reflect a causal pathway. Furthermore, the epidemiological relevance of WBC count as a marker of environmental-induced inflammation is supported by recent population-based findings; Lee and Yoon [15] reported that exposure to various ambient air pollutants is significantly associated with elevated WBC counts in the South Korean population. Collectively, the available evidence supports WBC as an informative, albeit non-specific, indicator of systemic inflammation potentially responsive to environmental exposures. Collectively, these findings—spanning from laboratory experiments to population-level observations—suggest that mixed VOC exposure patterns may be linked to systemic inflammatory responses, though longitudinal studies are needed to establish temporality and causation.

Smoking emerged as a strong independent determinant of both urinary VOC metabolite patterns and WBC levels. Epidemiological studies have consistently demonstrated that cigarette smoking is associated with elevated WBC counts, reflecting smoking-induced leukocytosis that is at least partially reversible following smoking cessation [18,19]. Among non-smokers, neither the solvent-dominant nor the combustion-dominant index was significantly associated with WBC count. In contrast, among smokers, both indices were positively associated with WBC levels. These findings emphasize that the health impacts of source-oriented VOCs should be interpreted within the context of individual lifestyle factors, such as smoking, rather than as a uniform effect across the entire population.

Our findings align with earlier clinical and population-based studies—including those conducted in Asian populations [20]—which have consistently reported elevated leukocyte counts in smokers, further reinforcing the well-established role of cigarette smoking in inducing systemic inflammation [29,30].

Importantly, we observed a significant interaction between the urinary VOC exposure index and smoking status, indicating that the association between solvent-dominant VOC exposure and WBC count was stronger among smokers than non-smokers. Although the cross-sectional design limits mechanistic interpretation, several plausible biological explanations may account for this effect modification. First, cigarette smoking may prime inflammatory pathways through chronic activation of oxidative stress responses, potentially increasing susceptibility to additional pro-inflammatory stimuli from environmental VOC exposure [12]. Second, smoking is known to induce cytochrome P450 enzymes involved in xenobiotic metabolism, which may alter the metabolic activation or detoxification of solvent- dominant VOCs, thereby modulating their inflammatory effects [31]. Third, smokers experience higher combined VOC burdens from both tobacco smoke and environmental sources, which may exceed a threshold for detectable inflammatory responses more readily than either source alone. Taken together, these results suggest that host susceptibility factors—particularly smoking status—may play a more critical role in determining VOC-related inflammatory responses than strict source categorization alone. Notably, the main-effect coefficients for the two indices were similar in magnitude, suggesting modest overall effect sizes and limited evidence of materially different impacts by source classification.

These potential mechanisms, while speculative in the context of a cross-sectional study, are consistent with experimental evidence and underscore the importance of explicitly evaluating effect modification in mixture exposure studies. Rather than acting solely as a confounder, smoking appears to modify susceptibility to VOC-related inflammatory effects, with important implications for risk assessment and targeted intervention strategies.

From a public health perspective, these findings highlight the relevance of considering smoking status when evaluating inflammation-related impacts of indoor VOC mixtures. However, longitudinal studies are needed to establish causality before specific intervention recommendations can be made.

This study has several notable strengths, including the use of nationally representative biomonitoring data from KNHANES, a source-oriented mixture approach using factor analysis to characterize real-world co-exposure patterns, environmental validation of urinary biomarker patterns through associations with indoor airborne VOC measurements, and explicit evaluation of effect modification by smoking status rather than merely adjusting for smoking as a confounder.

Several limitations should be considered. First, the cross-sectional design of KNHANES limits causal inference and precludes assessment of temporal relationships between VOC exposure and inflammation; longitudinal studies are needed to establish whether VOC exposure precedes inflammatory changes. Second, WBC count is a non-specific inflammatory marker, and the absence of additional biomarkers such as C-reactive protein, cytokines, or differential leukocyte counts restricts mechanistic interpretation and the ability to identify specific inflammatory pathways. Third, urinary VOC metabolites reflect relatively short-term exposure (hours to days) and may not capture long-term cumulative exposure patterns or chronic health effects. Fourth, although we adjusted for multiple potential confounders, residual confounding by unmeasured occupational exposures, dietary factors, or other environmental chemicals cannot be excluded. Finally, the generalizability of our findings to other populations with different exposure profiles or genetic backgrounds remains uncertain.

Future longitudinal studies incorporating repeated exposure measurements and multiple inflammatory biomarkers are warranted to further elucidate the role of mixed VOC exposure in systemic inflammation and related health outcomes.

## 5. Conclusions

In this nationally representative study of Korean adults, a solvent-dominant urinary VOC exposure pattern was positively associated with white blood cell count. Both urinary VOC exposure indices were significantly associated with the airborne VOC exposure index, supporting their relevance as internal markers of indoor VOC exposure. When effect modification was considered, both urinary VOC exposure indices were associated with elevated WBC count among smokers but not among non-smokers. These findings suggest that cigarette smoking may modify the association between urinary VOC exposure and systemic inflammation. These findings underscore the importance of considering host susceptibility factors, such as smoking status, in the risk assessment of mixed VOC exposure in indoor environments.

## Figures and Tables

**Table 1:** General characteristics of the study population.

Variable	Total	Male	Female	*p* Value ^a^
N	1859	844	1015	
Age (years), mean ± SD	54.17 ± 17.00	54.24 ± 16.99	54.10 ± 17.02	0.862
BMI (kg/m^2^), mean ± SD	24.01 ± 3.67	24.73 ± 3.47	23.42 ± 3.73	<0.0001
Smoking status, n (%)				
No	1575 (85.00)	597 (70.99)	978 (96.64)	<0.0001
Yes	278 (15.00)	244 (29.01)	34 (3.36)	
Alcohol consumption, n (%)				
No	919 (49.60)	288 (34.24)	631 (62.35)	<0.0001
Yes	934 (50.40)	553 (65.76)	381 (37.65)	
Physical activity, n (%)				
No	1011 (57.48)	438 (55.03)	573 (59.50)	0.145
Yes	748 (42.52)	358 (44.97)	390 (40.50)	
Education, n (%)				
≤Elementary School	330 (18.72)	97(12.17)	233 (24.12)	<0.0001
Middle School	168 (9.53)	72 (9.03)	96 (9.94)	
High School	553 (31.37)	285 (35.76)	268 (27.74)	
≥College	712 (40.39)	343 (43.04)	369 (38.20)	

SD, Standard Deviation. ^a^ *p* values were obtained using Student’s *t*-test for continuous variables and chi-square test for categorical variables.

**Table 2:** Rotated factor loadings of urinary VOC metabolites identified by principal component analysis.

Biomarker	Parent Compound	Factor 1 (Combustion-Dominant)	Factor 2 (Solvent-Dominant)	Communality
SPMA	Benzene	**0.716**	−0.033	0.514
BMA	Toluene	**0.695**	−0.045	0.486
DHBMA	1,3-Butadiene	**0.694**	0.277	0.558
3HPMA	Acrolein	**0.589**	0.408	0.513
MA	Styrene	0.540	0.231	0.345
PGA	Ethylbenzene & Styrene	0.332	0.433	0.298
3,4-MHA	Xylene	0.018	**0.943**	0.890
2-MHA	Xylene	0.090	**0.928**	0.869

Factor analysis was performed on log-transformed urinary VOC metabolite concentrations using varimax rotation. Factor loadings ≥ 0.50 are shown in bold, and communality indicates the proportion of variance explained by the retained factors. BPMA was examined but not retained in the final exposure pattern due to its low communality.

**Table 3:** Associations between airborne VOC exposure index and urinary VOC exposure indices after adjustment for covariates.

Variable	Urinary Combustion-Dominant Index			Urinary Solvent-Dominant Index		
	β	95% CI	*p*-Value	β	95% CI	*p*-Value
Airborne VOC index	0.039	0.004, 0.075	0.032	0.241	0.189, 0.292	<0.001
Sex	−0.379	−0.439, −0.319	<0.001	−0.093	−0.179, −0.008	0.033
Age (years)	0.022	0.021, 0.024	<0.001	0.008	0.005, 0.010	<0.001
Smoking status	0.444	0.364, 0.525	<0.001	1.295	1.180, 1.410	<0.001
Alcohol consumption	0.027	0.031, 0.086	0.357	0.060	−0.023, 0.143	0.157
BMI (kg/m^2^)	−0.007	−0.015, 0.001	0.055	−0.016	−0.027, −0.005	0.003
Indoor CO_2_ (ppm)	−0.001	−0.001, −0.001	<0.001	−0.001	−0.001, −0.001	0.002

Values are regression coefficients (β) with 95% confidence intervals (CI) derived from generalized linear regression models. The airborne VOC exposure index represents a factor-derived index based on indoor airborne VOC measurements. Models were adjusted for age, sex, body mass index, smoking status, alcohol consumption, diabetes status, and indoor carbon dioxide (CO_2_) concentration.

**Table 4:** Associations between urinary VOC exposure indices and white blood cell count.

Variable	Model 1: Solvent-Dominant Index			Model 2: Combustion-Dominant Index		
	β	95% CI	*p*-Value	β	95% CI	*p*-Value
Urinary VOC index	0.091	0.009, 0.172	0.030	0.107	−0.013, 0.226	0.081
Sex	0.300	0.143, 0.456	0.001	0.332	0.169, 0.495	<0.001
Age (years)	−0.003	−0.008, 0.001	0.127	−0.005	−0.010, −0.000	0.045
Smoking status	0.929	0.694, 1.164	<0.001	1.001	0.784, 1.217	<0.001
Alcohol consumption	−0.183	−0.335, −0.032	0.018	−0.181	−0.333, −0.030	0.019
Diabetes	0.442	0.193, 0.692	0.001	0.433	0.183, 0.684	0.001
Body mass index (kg/m^2^)	0.082	0.062, 0.101	<0.001	0.081	0.062, 0.101	<0.001

Regression coefficients (β) represent changes in white blood cell count per one–standard deviation increase in the urinary VOC indices (combustion-dominant or solvent-dominant). Female, non-smoker, non-drinker, and non-diabetic participants were used as reference categories. Models were adjusted for age, sex, body mass index, alcohol consumption, smoking status, and diabetes status.

**Table 5:** Interaction between Urinary VOC exposure indices and smoking status in relation to WBC count.

	Non-Smoker			Smoker			*p* for Interaction
	β	95% CI	*p*-Value	β	95% CI	*p*-Value	
Solvent-dominant index	−0.027	−0.117, 0.063	0.557	0.571	0.391, 0.752	<0.001	<0.001
Combustion-dominant index	−0.023	−0.152, 0.106	0.728	0.614	0.380, 0.849	<0.001	<0.001

β represents the change in WBC count per one standard deviation (SD) increase in each urinary VOC exposure index (solvent-dominant and combustion-dominant). β values for smokers were calculated as β_non-smoker + β_interaction. All models were adjusted for age, sex, BMI, alcohol consumption, and diabetes status.

## Data Availability

The data that support the findings of this study are openly available through the Korea National Health and Nutrition Examination Survey (KNHANES) data sharing service (https://knhanes.kdca.go.kr, accessed on 15 January 2026), subject to data use approval.

## Abbreviations

SPMA	S-phenylmercapturic acid (N-acetyl-S-(phenyl)-L-cysteine)
BMA	benzylmercapturic acid (N-acetyl-S-(benzyl)-L-cysteine)
PGA	phenylglyoxylic acid
MA	mandelic acid
2-MHA	2-methylhippuric acid
3- and 4-MHA	3- and 4-methylhippuric acids
DHBMA	N-acetyl-S-(3,4-dihydroxybutyl)-L-cysteine
3-HPMA	N-acetyl-S-(3-hydroxypropyl)-L-cysteine
Bpma	1-Bromopropane
HCHO	Formaldehyde
